# Remarks on Goitre, with Report of a Case

**Published:** 1895-12

**Authors:** Joseph A. White

**Affiliations:** Richmond, Va.; Professor of Ophthalmology, and Associate Professor of Otology and Laryngology, University College of Medicine


### REMARKS ON GOITRE, WITH REPORT OF A CASE.

By JOSEPH A. WHITE, A.M., M.D., RICHMOND, VA.

Professor of Ophthalmology, and Associate Professor of Otology and Laryngology, University College of Medicine.

John Beatie, aged forty-six, a stonecutter by trade, consulted me in December, 1894, about his eyes. I prescribed the needed glasses, and noticed he had a very large goitre, which I took to be a fibro-cystic enlargement of the thyroid gland, from a very cursory examination. There were several cysts, one in the right and two in the left lobe of the gland. I asked why he did not have it treated, and his reply was, that he was so discouraged by the failures in the past that he had given up, although it was steadily increasing in size. It had begun about fifteen years previous, as far as he could recollect, and for over twelve years had been a marked deformity. He had tried iodine injections, tapping sac with an aspirator, the constant current, and, as he expressed it, the "electric needle."

On June 27, 1895, he returned and asked me what could be done with the goitre, as it was becoming an impediment to respiration, because of compression of the windpipe. Having had very satisfactory results with iodide of potassium, by cataphoresis in goitrous cases, I commenced treatment with this plan. Up to Wednesday, July 27th, I had used cataphoresis twelve times with some slight diminution of the tumor and improvement to respiration. On July 17th I opened the central cyst at the bottom, and drained off a quantity of dark, brownish, muddy fluid, similar to what is seen in ovarian cysts, etc. The sac was washed out until the returning liquid was clear, and a small quantity of iodine injected. The opening was kept free by packing with gauze. For several days the sac was washed out and packed daily with considerable improvement. On July 27th I cut into the left cyst from the top, and passed the knife across and out at the opening made in the lower part of the central cyst, which was followed by a discharge of the same fluid and venous hemorrhage. Iodine was injected, and a silver drainage wire was inserted, entering one opening and out through the other. Antiseptic gauze was packed into the cyst. The next day he was unable to come to my office, being confined to bed. He was visited twice daily; the sac was washed out with peroxide of hydrogen and bichloride of mercury. On the 31st his neck was enormously swelled, especially on the right side—all the lymphatics being involved—and deglutition was almost entirely arrested. I at once cut into the right cyst and drew off a great quantity of fluid. This sac was connected with the others, and a drainage wire, with packing, inserted. Every day the sacs were washed out, disinfected, and packed. Quinine and stimulants were used internally. On August 4th his temperature rose to 104°, pulse 126; and on the 5th and 6th, 101°; and in a few days normal. He was then put on potash, which was increased to sixty grains three times a day. By September his neck was reduced almost to its normal size. Some little thickening of the parenchyma of the gland remained, although the cyst had entirely disappeared. For this cataphoresis was continued, and you saw his condition two weeks ago, when he presented himself to this academy. From wearing a seventeen-inch collar, he was wearing a fourteen and a half inch. The contour of the neck was perfectly natural. The thyroid cartilage was prominently defined, and, with the exception of the scars of the incision, there was little or no sign of trouble of the thyroid gland.

The above case is an interesting one in its results, and on account of the prominence which the thyroid gland has assumed in medical journals the last year or so, especially in relation to the use of the extract of the thyroid glands of animals in the treatment of myxedema; although the subject of goitre itself seems to have received but little attention. I find, in looking over the medical journals of the last year or so, that very little is said in relation to this matter; most references to the thyroid gland being in connection with the treatment of myxedema. Goitre, however, whilst receiving but a short chapter in most text-books on surgery, is a subject of considerable importance. We have different troubles of the thyroid gland, such as acute thyroiditis, bronchocele, or goitre of different forms, such as follicular, fibrous, fibro-cystic, and cystic; and that peculiar complex of symptoms, known as exophthalmic goitre.

I have no doubt that the reason that we only occasionally find writings upon this subject in the regular medical journals, is due to the lack of knowledge of the functions of the thyroid gland, and the difficulty of explaining, etiologically, the various changes that take place. Some recent investigations into the functions of the gland by Hurthle, Eulenberg, and others may result in a better understanding of the pathological alterations. Hurthle reports that the colloid substance in the follicles is produced by the protoplasm in the epithelial cells, and that the secretion of the gland consists in the formation of this colloid substance. It is supposed that pathological changes in the gland are due to some deterioration of this normal secretion. Probably, some evidence of this is found in the fact that the same treatment (the use of thyroid extract, for example) decreases the enlarged gland and improves the bad results which come from the absence of it, such as myxedema. Another corroboration, probably, is the well-known fact that cretinism is found in connection with both hypertrophy and atrophy of the gland. Eulenberg thinks that the constitutional symptoms of exophthalmic goitre may be the direct toxic effects of absorption into the veins of the increased altered secretion of the follicles, which produces chemical changes in the constitution of the blood. If this theory is correct, the nervous origin of exophthalmic goitre must be discarded. That it is tenable, is shown by the fact that nearly the same symptoms are produced by the artificial introduction of thyroid secretion in excess. But these theories in regard to the etiology of the pathological changes of the thyroid gland are somewhat speculative as yet, and require further investigation and confirmation.

The treatment of troubles of the thyroid has received lately considerable impetus. We might divide into medicinal, electrical, and operative.

*Medicinal.*—The medical treatment is by the internal administration of suggested remedies, such as iodide of potash, fluoric acid, thyroid extract, etc., and locally, by the introduction in the substance of the gland of iodine, iodoform, etc. Every one is familiar with the iodine treatment. It is the oldest, and has held its ground longer than any other, with varying success. Probably 90 per cent. of follicular and fibro-cystic goitres are reduced in volume by this treatment, but few radical cures are recorded. Garé, of Tübingen, reports, however, very great success with the injection of iodoform, one part to seven of oil and ether. Kocher, of Berne, has used thyroid extract in twelve cases, all of which were improved, some cured. Bruns, of Tübingen, also tried feeding, in twelve cases, with fresh calf thyroid. Four or five cases were cured, and the others, with the exception of three, much improved.

*Electrical.*—Under this heading I would include three methods of using electricity. First, by galvanism, passing the continuous current through the gland, both poles being on the tumor; second, by electrolysis; and third, by cataphoresis. With the use of the constant current I have had little or no experience. It has been suggested for the reduction of the different kinds of glandular enlargements, and has been used with varying success. Electrolysis I have used in follicular and fibrous goitre. The negative pole is generally passed into the growth, and a current of ten milliampères is turned on and continued for about five or ten minutes. This can be repeated in from three days to a week, according to the amount of irritation set up; the strength of the current being increased until we can use as much as forty or fifty milliampères. Gradual reduction of the growth takes place under this treatment, and a number of cures are recorded as its results. It is of very little service, however, in cystic goitre, because we cannot get the effect of the negative pole in the alteration of the tissue of the growth as we do in the more solid tumors. It has been suggested to tap the cyst, wash it out, fill it to distention with chloride of sodium solution, and, by this means, receive the full effects. With electrolysis, from four or five to a dozen or more sittings are required.

*Cataphoresis.*—The third method, that of cataphoresis or the introduction of remedies by the direction of the electric current, has been in my hand a very satisfactory treatment, particularly in follicular goitre. I can record two or three cases in the last eighteen months where I have had the most satisfactory results from the use of iodide of potash by this method. I use, attached to the positive pole, a metal disc which is covered with wet chamois or cotton, upon which is packed as much powdered iodide of potash as it will hold. This is covered over with a thin pledget of wet cotton and applied to the growth. The negative pole is held in the hand, or applied to the back of the neck, or between the shoulder blades. I have seen very little notice of this method of treatment. The only case that I know of recorded was one reported by Dr. McGuire two or three years ago in the *Virginia Medical Monthly.* In the goitrous enlargement of bronchocele, which one observes in young people, young girls especially, about the time of puberty, I don't know any more satisfactory treatment. It is true that this form of bronchocele occasionally manifests itself only at the time, or during the period of, menstruation, and very frequently gets well of itself. I am not, however, referring to this form, but to those cases of persistent enlargement of the gland, which not only is seen during the menstrual period, but is present more or less all the time until active measures are instituted for its relief. I have seen cases of follicular bronchocele that have become very large in women because no attention was paid to it in the stage where it would swell up and go down, as it were, on the theory that it would get well of itself. One of these was very large, and persisted for several years, and was cured by the application of iodide of potash by cataphoresis. This case I have already mentioned, and it is one known to the most of you. I am satisfied that further investigation into this method of applying remedies will show it to be of great value. In regard to operation, I am satisfied that in cutting open the cysts, as I did in the case above recorded, that I was exposing this patient to as great danger as if I had removed part of the gland. This was evidenced by the symptoms that developed.

Partial thyroidectomy, or strumectomy as some call it, is recommended by many authors for follicular and fibrous goitre. Strom, of Christiana, has reported quite a number of operations with success. He also advises enucleation of the cyst for cystic goitre.

Morris, in the *Lancet*, January 5, 1895, reported two cases of multiple cyst of the thyroid gland, such as the case before reported, in which he also practiced incision with satisfactory success.

Marsh, in the *Birmingham Medical Review*, reported five cases of bronchocele, operated on for urgent pressure symptoms (all the cases being of comparatively short duration), in which he had good results, and he advises removal of the isthmus and as much of the lateral lobes as may be needed to relieve pressure, which is followed by atrophy of the rest of the gland.

Brooks reports two cases of partial thyroidectomy followed by success.

Operation has also been suggested and performed by quite a number of authors for exophthalmic goitre, on the ground that it is hyperplasia of the gland structure, and that the nervous symptoms are due to the toxic effects of the altered secretions, and a number of cures are reported. Greenfield's article in the *British Medical Journal*, December, 1893, is probably the best of the contributions. All operators, however, have come to one conclusion—that complete removal of the glands is unjustifiable, and that all operations are more or less dangerous, death on the table having resulted in a number of cases from collapse. It is doubtful if any deaths have resulted from hemorrhage, although in some cases the bleeding is hard to control, because of the difficulty of applying ligatures to the vessels whose walls are in such a condition that ligatures will tear loose. To arrest the bleeding by packing is not satisfactory, and may be dangerous. I have no doubt that during operations on the thyroid gland, some of the fatal results were due to prolonged pressure on the pneumogastric nerve. If proper care is taken in performing the operation, the bleeding arrested as the operation proceeds, and the field kept as aseptic as possible, I believe the operation of partial thyroidectomy would be comparatively safe. Care must also be taken not to injure the recurrent laryngeal nerve. This operation has been successful in a great many instances, but there have been some failures—as is the case with all operations in surgery.

200 *East Franklin street.*

